# Supplementary material for: A comprehensive analysis of canonical biological pathways linking milk yield and quality traits to key fertility indicators in Murciano-Granadina dairy does
Source: PLoS One. 2026 Apr 29;21(4):e0348264. doi: 10.1371/journal.pone.0348264 (PMC13127934; doi:10.1371/journal.pone.0348264)
Supplement: S2 Material — (DOCX) [file pone.0348264.s002.docx]

**Material S2.** Canonical functions F1 and F2 describing multivariate associations between fertility indices and milk production and composition traits.

**Canonical function 1 (F1):** Energy Balance–Production–Fertility Trade-Off Pathway

**Fertility-side variate (U1):**

U1 = −0.030 × Fertility per day of insemination − 0.980 × Fertility per buck batch and day of insemination

**Milk composition-side variate (V1):**

V1 = 1.030 × Milk Yield − 0.016 × Fat (%) + 0.279 × Protein (%) − 0.291 × Lactose (%) + 0.645 × Dry Matter (%) + 0.285 × Somatic Cells − 0.529 × Milk Yield 150d + 0.228 × Lactose 150d − 0.964 × Dry Matter 150d

On the fertility side, fertility per buck batch and day of insemination dominates negatively, indicating that U1 contrasts high versus low fertility across batches and insemination days. On the milk composition side, milk yield and dry matter content load strongly positive, whereas standardized dry matter at 150 days and lactose show negative weights. Therefore, F1 represents a gradient linking higher milk yield and dry matter content with lower fertility per buck batch and day, highlighting potential trade-offs between production and reproductive performance.

**Canonical function 2 (F2): Endocrine Integration and Lactation-Timing Pathway**

**Fertility-side variate (U2):**

U2 = −1.375 × Fertility per day of insemination + 0.965 × Fertility per buck batch and day of insemination

**Milk composition-side variate (V2):**

V2 = 0.373 × Milk Yield + 0.595 × Fat (%) + 0.118 × Protein (%) − 0.283 × Lactose (%) − 0.700 × Dry Matter (%) − 0.269 × Somatic Cells + 0.049 × Milk Yield 150d − 0.273 × Lactose 150d + 0.999 × Dry Matter 150d

U2 is dominated by fertility per day of insemination negatively and fertility per buck batch positively, contrasting daily versus batch fertility. V2 is strongly influenced by standardized dry matter at 150 days positively and dry matter % negatively, reflecting variation in milk composition across the lactation period. F2 therefore links an axis of daily versus batch fertility patterns with milk composition traits, particularly highlighting the role of dry matter distribution over lactation.
