# Supplementary material for: A comprehensive analysis of canonical biological pathways linking milk yield and quality traits to key fertility indicators in Murciano-Granadina dairy does
Source: PLoS One. 2026 Apr 29;21(4):e0348264. doi: 10.1371/journal.pone.0348264 (PMC13127934; doi:10.1371/journal.pone.0348264)
Supplement: S3 Table — (DOCX) [file pone.0348264.s008.docx]

**Table S3.** Results for the tests of equality of group means to test for difference in the means across fertility rate scale levels once redundant variables have been removed.

| Fertility rate scales | Variable | Unstandardized Milk Yield | Unstandardized Fat | Unstandardized Protein | Unstandardized Lactose | Unstandardized Dry Matter | Unstandardized Somatic Cells Count | Standardized Milk Yield 150 days | Standardized Lactose 150 days | Standardized Dry Matter 150 days | Semen Type - Fresh | Semen Type - Frozen |
| --- | --- | --- | --- | --- | --- | --- | --- | --- | --- | --- | --- | --- |
| Fertility per day of insemination | Wilks' Lambda | 0.996 | 0.998 | 0.998 | 1.000 | 0.999 | 0.998 | 0.999 | 0.999 | 0.995 | 0.970 |  |
|  | F | 33.750 | 14.770 | 16.813 | 3.109 | 10.390 | 16.513 | 5.941 | 11.950 | 41.160 | 253.820 |  |
|  | DF1 | 4 | 4 | 4 | 4 | 4 | 4 | 4 | 4 | 4 | 4 | 4 |
|  | DF2 | 32688 | 32688 | 32688 | 32688 | 32688 | 32688 | 32688 | 32688 | 32688 | 32688 | 32688 |
|  | p-value | < 0.0001 | < 0.0001 | < 0.0001 | 0.014 | < 0.0001 | < 0.0001 | < 0.0001 | < 0.0001 | < 0.0001 | < 0.0001 |  |
| Fertility per buck batch and day of insemination | Wilks' Lambda | 0.996 | 0.999 | 0.999 | 0.999 | 1.000 | 0.999 | 0.999 | 0.999 | 0.999 | 0.986 |  |
|  | F | 35.294 | 4.432 | 5.987 | 5.000 | 2.305 | 8.775 | 4.974 | 7.957 | 6.743 | 114.794 |  |
|  | DF1 | 4 | 4 | 4 | 4 | 4 | 4 | 4 | 4 | 4 | 4 | 4 |
|  | DF2 | 32688 | 32688 | 32688 | 32688 | 32688 | 32688 | 32688 | 32688 | 32688 | 32688 | 32688 |
|  | p-value | < 0.0001 | 0.001 | < 0.0001 | 0.001 | 0.056 | < 0.0001 | 0.001 | < 0.0001 | < 0.0001 | < 0.0001 |  |
| Fertility per day by semen type | Wilks' Lambda | 0.992 | 0.999 | 0.997 | 1.000 | 0.999 | 0.998 | 0.999 | 0.998 | 0.995 | 0.964 |  |
|  | F | 58.015 | 8.457 | 21.541 | 3.267 | 5.272 | 17.749 | 10.458 | 13.990 | 33.805 | 276.109 |  |
|  | DF1 | 4 | 4 | 4 | 4 | 4 | 4 | 4 | 4 | 4 | 4 | 4 |
|  | DF2 | 29385 | 29385 | 29385 | 29385 | 29385 | 29385 | 29385 | 29385 | 29385 | 29385 | 29385 |
|  | p-value | < 0.0001 | < 0.0001 | < 0.0001 | 0.011 | < 0.0001 | < 0.0001 | < 0.0001 | < 0.0001 | < 0.0001 | < 0.0001 |  |
