# Supplementary material for: A comprehensive analysis of canonical biological pathways linking milk yield and quality traits to key fertility indicators in Murciano-Granadina dairy does
Source: PLoS One. 2026 Apr 29;21(4):e0348264. doi: 10.1371/journal.pone.0348264 (PMC13127934; doi:10.1371/journal.pone.0348264)
Supplement: S4 Table — Positive and negative values indicate the relative contribution of each trait to the canonical variable, with fresh semen consistently dominating F1. (DOCX) [file pone.0348264.s009.docx]

**Table S4.** Standardized canonical coefficients for the relationship between milk production traits (milk yield, fat, protein, lactose, dry matter, somatic cell count, and standardized 150-day measures) and fertility (per day of insemination, per buck batch/day, and per semen type) across four canonical functions (F1–F4). Positive and negative values indicate the relative contribution of each trait to the canonical variable, with fresh semen consistently dominating F1.

| Fertility Rate Scales | Fertility per day of insemination | | | | Fertility per buck batch and day of insemination | | | | Fertility per day by semen type | | | |
| --- | --- | --- | --- | --- | --- | --- | --- | --- | --- | --- | --- | --- |
| Functions | F1 | F2 | F3 | F4 | F1 | F2 | F3 | F4 | F1 | F2 | F3 | F4 |
| Unstandardized Milk Yield | -0.565 | 0.231 | 0.145 | 0.021 | -0.665 | 0.573 | 0.095 | 0.493 | -0.599 | 0.283 | 0.316 | -0.066 |
| Unstandardized Fat | 0.030 | -0.278 | -0.782 | 0.307 | -0.043 | -0.107 | 0.397 | -0.594 | 0.054 | 0.087 | -0.901 | 0.184 |
| Unstandardized Protein | -0.186 | -0.054 | 0.537 | 0.496 | -0.192 | 0.145 | -0.057 | 0.009 | -0.209 | -0.255 | 0.408 | 0.795 |
| Unstandardized Lactose | 0.170 | 0.254 | -0.050 | -0.016 | 0.212 | -0.007 | -0.134 | -0.400 | 0.159 | 0.291 | -0.029 | -0.054 |
| Unstandardized Dry Matter | -0.285 | 0.921 | 0.347 | 0.227 | -0.200 | -0.100 | -0.175 | 0.453 | -0.184 | 1.057 | 0.316 | -0.134 |
| Unstandardized Somatic Cells Count | -0.177 | 0.240 | -0.082 | 0.307 | -0.140 | 0.632 | -0.254 | -0.253 | -0.188 | 0.177 | -0.060 | 0.498 |
| Standardized Milk Yield 150 days | 0.443 | -0.006 | -0.465 | -0.386 | 0.343 | -0.610 | -0.275 | 0.197 | 0.377 | 0.108 | -0.401 | 0.046 |
| Standardized Lactose 150 days | -0.181 | -0.251 | 0.766 | -0.287 | -0.117 | 0.495 | 0.816 | 0.555 | -0.132 | -0.328 | 0.684 | -0.047 |
| Standardized Dry Matter 150 days | 0.494 | -1.270 | -0.370 | -0.271 | 0.359 | -0.324 | 0.230 | -0.060 | 0.325 | -1.335 | -0.446 | -0.110 |
| Semen Type - Fresh | 0.827 | 0.318 | 0.142 | 0.239 | 0.786 | 0.363 | -0.103 | 0.197 | 0.834 | 0.277 | 0.181 | 0.270 |
| Semen Type - Frozen | 0.000 | 0.000 | 0.000 | 0.000 | 0.000 | 0.000 | 0.000 | 0.000 | 0.000 | 0.000 | 0.000 | 0.000 |
