# Supplementary material for: A comprehensive analysis of canonical biological pathways linking milk yield and quality traits to key fertility indicators in Murciano-Granadina dairy does
Source: PLoS One. 2026 Apr 29;21(4):e0348264. doi: 10.1371/journal.pone.0348264 (PMC13127934; doi:10.1371/journal.pone.0348264)
Supplement: Attachment — Submitted filename: review-plos one does granadina.docx [file pone.0348264.s010.docx]

I have carefully evaluated the manuscript titled "A Comprehensive Analysis of Canonical Biological Pathways Linking Milk Yield and Quality Traits to Key Fertility Indicators in Murciano-Granadina Dairy Does" Below are my valuable comments:

Firstly, I would like to appreciate that the study employed a comprehensive methodology with sophisticated statistics, integrating CCA, RCCA, and CHAID. All the statistical analyses were also validated. This represents a clear strength of the manuscript. However, major revisions will be required to improve the manuscript's clarity, focus, and defensibility.

The areas requiring improvement are:

1. **The length of the manuscript**

The manuscript is excessively long and requires condensation. It drives the reader away from the main conclusion of the manuscript, especially for readers who are not statisticians. The presence of numerous equations in the methodology, as well as canonical functions in the results section, may overwhelm readers. Consider moving all the equations and the full formula for canonical functions to a single supplementary material. Every symbol should be explained in the supplementary material. Include only the most informative equation and the key findings in the main text. The result is quite lengthy and could be condensed for clarity. Combine the results of canonical dimensions, Bartlett’s statistics, Wilks' lambda, and Pillai’s trace into one concise subsection, naming it 'Dominance of the First Canonical Axis'. Avoid repeating the same information, such as F1 explains most variance, semen type dominates, and milk yield and dry matter influence fertility in CDA, RCCA, in clustering, and in CHAID, as well as in the Discussion. This increases the manuscript's length without providing any additional insight. The result section is occasionally found to be written as a discussion section, which should be shifted under the discussion section.

2. **The weak effect sizes are over-interpreted**

The results in the manuscript are

• Canonical correlation = **0.132**

• Redundancy = **1–2%**

• Pearson correlations = **range from 0.02 to 0.08,**

**which are statistically significant, but the effect sizes are weak. The phrases should be changed. Instead of writing milk compositions determine or influence fertility, it should be phrased as Milk composition is modestly associated with fertility. Also, include limitations acknowledging this, stating that fertility is only weakly predictable from milk traits and genetics, seasons, management, and health likely explain a greater variance.**

3. **Experimental design should be clearly demonstrated**
a. **The experimental design should be clearer and understandable so that the work can be reproduced by others. Include a flowchart diagram explaining the many steps in a workflow, from sampling and measurements, preliminary data checks, multivariate analysis, to data interpretation, including all the names of the statistical tests, how they were employed one after another, and the validation steps.**
b. **Many statistical methods were used, including CDA, RCCA, correlation analysis, clustering, and CHAID decision trees. However, the purpose of the statistical methods is not explained enough, such as which test was exploratory and which was confirmatory. which tests the main hypothesis, and which test is supportive. The paper is statistically very impressive but conceptually diffuse.**
c. **Also, more questions should be addressed in the manuscript, such as**
1. **How were the female goats (does) selected? Were there any inclusion or exclusion criteria**
2. **How were the milk samples collected, their timing, frequency and method?**
3. **How were their fertility outcomes measured?**
4. **How were the data split across analyses, such as in CDA, RCCA, CHAID, and clustering?**
5. **Were there any interventions?**
4. **The categorisation of the fertility should be justified**

**The fertility was categorised from very low to very high without any biological justification. The authors need to explain how the cut points were defined.**

**Recommendation: Include references from industrial standards or historical fertility benchmarks, or mention that the cut-offs correspond to extreme tails of the observed fertility distribution. This avoids arbitrariness.**

5. **The Discussion did not contain any additional information on Semen type, as it was the strongest discriminator**

**It must be mentioned that what made the semen type show such a strong effect. It might not only be due to biological reasons, such as damaged sperm due to freezing. Possible reasons might also include mishandling procedures, such as incorrect temperature during thawing, transport, or storage, etc.**
