# Supplementary material for: A comprehensive analysis of canonical biological pathways linking milk yield and quality traits to key fertility indicators in Murciano-Granadina dairy does
Source: PLoS One. 2026 Apr 29;21(4):e0348264. doi: 10.1371/journal.pone.0348264 (PMC13127934; doi:10.1371/journal.pone.0348264)
Supplement: Attachment — Submitted filename: Response to Reviewers (3rd round).docx [file pone.0348264.s012.docx]

**Francisco Javier Navas González**

Department of Genetics, Faculty of Veterinary Sciences

University of Córdoba

Rabanales University Campus, 14071

Córdoba (Spain)

+34 651679262

fjng87@hotmail.com

19/02/2026

Dear Editor,

All the team responsible for this paper acknowledge the comments from the reviewers and editor, as they help to improve the quality of our manuscript. In the following paragraphs, we will describe and address how referees’ new recommendations were followed. A point-by-point response to comments is provided as well as a file where changes are highlighted

1. In the online submission form, you indicated that "Data will be made available from the corresponding author F.J.N.G. upon reasonable request."

   All PLOS journals now require all data underlying the findings described in their manuscript to be freely available to other researchers, either
   1. In a public repository,
   2. Within the manuscript itself, or
   3. Uploaded as supplementary information.

   This policy applies to all data except where public deposition would breach compliance with the protocol approved by your research ethics board. If your data cannot be made publicly available for ethical or legal reasons (e.g., public availability would compromise patient privacy), please explain your reasons on resubmission and your exemption request will be escalated for approval.

   2. When completing the data availability statement of the submission form, you indicated that you will make your data available on acceptance. We strongly recommend all authors decide on a data sharing plan before acceptance, as the process can be lengthy and hold up publication timelines. Please note that, though access restrictions are acceptable now, your entire data will need to be made freely accessible if your manuscript is accepted for publication. This policy applies to all data except where public deposition would breach compliance with the protocol approved by your research ethics board. If you are unable to adhere to our open data policy, please kindly revise your statement to explain your reasoning and we will seek the editor's input on an exemption. Please be assured that, once you have provided your new statement, the assessment of your exemption will not hold up the peer review process.

Response: As stated in the previous round of revisions” Data is either included as a part of the manuscript or has been uploaded as supplementary information. Updated information can also be downloaded from the Spanish National System of Breed Information (ARCA) Public Repository.

https://www.mapa.gob.es/es/ganaderia/temas/zootecnia/razas-ganaderas/razas/catalogo-razas/caprino/murciano-granadina/”

3. Please note that PLOS ONE is unable to publish previously copyrighted maps or satellite images, or images created using proprietary data. For these reasons, we cannot publish images generated by software which copyrights their output (such as Google Maps, Street View, and Earth). In order to use these images in your submission, we require explicit permission from the copyright owner to publish the figures under the CC BY 4.0 license.

At this time, please kindly clarify the following regarding Figure 2:

a) Where did the authors obtain the maps, basemaps, shapefiles, map data, etc. in Figure 2?

b) Please state whether the maps, basemaps, shapefiles, map data, etc. have been previously copyrighted to your knowledge.

c) If any of the maps, basemaps, shapefiles, map data, etc. in this image have been previously copyrighted, we require specific consent from the copyright holder to publish these images in PLOS ONE, under the CC BY 4.0 license.

Response: Maps were specifically created by corresponding author for this paper.

Sincerely,

To this end, we thank you for your time and attention and for considering this manuscript.

Should you require any further information do not hesitate to contact me via email or telephone at any time.

Yours faithfully,

Dr Fco. Javier Navas González
